# Are letter features weighted asymmetrically during the early moments of lexical access? Evidence from masked priming in Serbian Cyrillic

**DOI:** 10.3758/s13423-026-03006-2

**Published:** 2026-09-08

**Authors:** Olga Solaja, Manuel Perea

**Affiliations:** 1 ERI-Lectura and Department of Methodology, Universitat de València, Av. Blasco Ibáñez, 21, 46010 Valencia, Spain; 2 Centro Investigación Nebrija en Cognición, Universidad Nebrija, Madrid, Spain

**Keywords:** Visual word recognition, Orthographic processing, Visual similarity, Letter features

## Abstract

Models of visual word recognition assume that abstract letter identities are encoded from visual features; but the principles governing this mapping remain underspecified. In Latin-script languages, masked primes that omit a diacritic facilitate recognition almost as much as identity primes (e.g., facil → FÁCIL [easy]), whereas primes that add a diacritic produce much weaker facilitation (e.g., féliz → FELIZ [happy]). This asymmetry has been explained by the perceptual salience of diacritics, or by assuming that feature presence carries more weight than feature absence. We tested these alternatives in Serbian Cyrillic, which contains letter pairs that differ by a single integrated letter feature (e.g., Л – Љ; H – Њ). We conducted a masked priming lexical decision experiment in which a target stimulus could be preceded by an identity prime, a visually similar prime created by feature omission or feature addition, or a visually dissimilar control prime. For targets containing complex letters (Љ/Њ), omission primes were as effective as identity primes (e.g., кoницa → КOЊИЦA). For targets containing simple letters (Л/H), addition primes produced a substantial cost relative to identity primes (e.g., кoњaц → КOHAЦ). Thus, the addition–omission asymmetry reported for diacritics generalizes to letters that differ by an integrated visual feature. These findings favor a general account in which the presence of a feature is weighted more heavily than its absence during the earliest stages of orthographic processing.

## Introduction

Skilled reading relies on the rapid mapping of a noisy, highly variable visual input onto stable orthographic representations that, in turn, drive lexical access. This initial feature-to-letter mapping must generalize across irrelevabnt perceptual variation (size, font, case; e.g., table, Table, and TABLE) and preserve sensitivity to small differences that distinguish visually similar letters (e.g., distinguishing *c* from *e* in *car*-*ear* or *m* from *n* in *lime*-*line*). Understanding how early visual feature extraction gives rise to abstract letter identities, while preserving fine-grained discrimination among similar letters, is therefore essential for a comprehensive model of visual word recognition.

Most leading models of visual word recognition assume a progression from low-level features to increasingly abstract orthographic codes (Dehaene et al., 2005; Grainger et al., 2008). However, computational models of visual word recognition have focused more on how letter units activate lexical representations than on how visual features are mapped onto letter units (for discussion, see Davis, 2010; Snell, 2025). As Balota et al. (2006, p. 289) noted, models of visual word recognition “take a leap of faith” in moving from visual features to letter units.

Notably, visual similarity effects have been reported during the early stages of word recognition using Forster and Davis’ (1984) masked priming technique in lexical decision experiments. As the primes are presented very briefly (around 50 ms) and masked, the obtained effects are commonly taken to reflect early orthographic processing with minimal strategic influences (Grainger, 2008; Perea et al., 2018). Previous research has found that word response times for a word like *NEUTRAL* are faster when preceded by the prime *nevtral –* where the letter u was replaced with the visually similar letter v *–* than when preceded by the prime *neztral –* where the letter *u* was replaced with the visually different letter *z* (Marcet & Perea, 2017, 2018; for ERP evidence, see also Gutiérrez-Sigut et al., 2019; for evidence with the Reicher-Wheeler task, see also Lally & Rastle, 2023).

Critically, visual-similarity priming with diacritical letters is asymmetric. Primes in which a diacritic is omitted from the target letter (e.g., facil → FÁCIL [easy]) tend to be nearly as effective as identity primes, whereas primes in which a diacritic is added to a non-diacritic target letter (e.g., féliz → FELIZ [happy]) produce substantially weaker facilitation (see Benyhe et al., 2023; Marcet et al., 2020; Perea et al., 2020). This pattern has been reported regardless of phonological similarity (Benyhe et al., 2023) and holds for both consonants and vowels (for the consonants n/ñ in Spanish, see Marcet et al., 2020), suggesting that it does not have a phonological origin. In particular, Benyhe et al. (2023) examined this question directly in Hungarian, a language in which diacritics may signal phonetic contrasts in vowel length and vowel quality. For example, o/ó and u/ú only differ in vowel length (e.g., o-/o/, ó-/oː/; u-/u/, ú-/uː/), while a/á and e/é also involve differences in vowel quality (a-/ɒ/, á-/aː/; e-/ɛ/, é-/eː/). Experiment 1 focused on vowel length contrasts (e.g., o vs. ó), while Experiment 2 examined both vowel length and vowel quality contrasts (e.g., a vs. á). They found the same asymmetric pattern in both experiments. When the target word contained a diacritic (e.g., RÓKA [fox]), a visually similar prime without the diacritic (e.g., roka), facilitated recognition just as much as the identity prime (e.g., róka); in contrast, when the target word did not contain a diacritic (e.g., MOZI [cinema]), the identity prime mozi was more effective than the visually similar prime with an added diacritic (e.g., mózi). Furthermore, parallel evidence comes from masked priming lexical decision experiments with Korean Hangul, an alphasyllabic script whose featural letters are combined into syllable blocks (Bae et al., 2025).

One explanation of this asymmetry is that it reflects the perceptual salience of diacritical marks as features added outside the base letter (Marcet et al., 2020; Perea et al., 2020, 2021). In their view, diacritics function as extraneous marks superimposed on the base letter rather than as part of the letter’s intrinsic form, thereby rendering diacritical letter forms more salient. This produces asymmetric relations in line with Tversky’s (1977) similarity theory: a less salient stimulus (the plain base letter, which lacks the added mark) is perceived as more similar to its salient counterpart (the letter that has the extra superimposed diacritic) than the reverse. This salience account is supported by the fact that the asymmetry largely disappears when the diacritic is no longer an extra mark (e.g., using í, where the accent replaces the i’s dot rather than being added outside it; see Perea et al., 2021).

A broader alternative is that the asymmetry reflects how bottom-up feature evidence is weighted during letter identification. Present features may provide stronger positive evidence for a letter identity than absent features provide negative evidence against it. Under brief and noisy viewing conditions, it is plausible that a feature present in the target may fail to be encoded, whereas the spurious insertion of an additional diagnostic feature is less likely. Thus, feature omissions may yield degraded but still compatible evidence, whereas feature additions may provide evidence for a competing letter identity. This logic is compatible with noisy-channel approaches to word recognition (Kinoshita et al., 2021; Norris & Kinoshita, 2012), but it is not specific to them. It also fits naturally with feature-based connectionist models of letter recognition in which feature detectors activate candidate letter units and competition among letter units resolves ambiguity (e.g., see Rey et al., 2009).

The present experiment was designed to adjudicate between a diacritic-specific account, according to which the asymmetry depends on the special salience and visually detached character of diacritical marks (Perea et al., 2020), and a more general account according to which the asymmetry reflects a property of evidence accumulation at the orthographic front-end (e.g., Kinoshita et al., 2021). To distinguish between these accounts, we need a script in which the critical feature is integral to the letter form itself rather than realized as a detached diacritic. Serbian Cyrillic provides such a test. It contains letters such as Љ and Њ (/ʎ/and/ɲ/) that are single alphabetic units in the Cyrillic script. Crucially, the distinguishing feature in Љ and Њ is integrated into the letter form itself rather than realized as a detached mark above or below a base letter. As a result, the present manipulation compares single-letter forms that differ in an integrated stroke, not base letters with and without an external diacritic. If the asymmetry in masked priming effects persists with this manipulation, it can no longer be attributed to the special status of detached diacritical marks. As in the Latin script, Serbian Cyrillic allows the standard masked-priming format in which lowercase primes are followed by uppercase targets (e.g., a/A, б/Б, в/B, etc.). This manipulation reduces direct physical prime–target overlap; importantly, prior evidence indicates that masked identity priming is driven primarily by abstract orthographic overlap rather than by the visual similarity of lowercase and uppercase forms (e.g., see Bowers et al., 1998; Perea et al., 2014).

As in previous research, we used masked priming lexical decision to manipulate whether the prime differed from the target by a single-letter feature. We created omission primes in which the prime lacked a letter feature present in the target (e.g., кoницa → КOЊИЦA [cavalry], where the prime contains the simple letter н instead of the complex target letter њ) and addition primes in which the prime contained a feature absent in the target (e.g., кoњaц → КOHAЦ [thread], where the prime contains the complex letter њ instead of the simple target letter н). These were presented alongside identity primes (e.g., кoњицa → КOЊИЦA; кoнaц → КOHAЦ) and visually dissimilar control primes (e.g., кoдицa → КOЊИЦA; кoдaц → КOHAЦ).

Critically, the two competing accounts make different predictions. If the asymmetry in visual-similarity priming effects is specific to detached diacritics (Perea et al., 2020), then substitutions involving integrated letter features should behave like ordinary visually similar substitutions, with no marked difference between omission and addition. If, however, the asymmetry reflects a more general property of early orthographic coding (Kinoshita et al., 2021), omission primes should pattern with identity primes for targets containing complex letters, whereas addition primes should be less effective for targets containing simple letters.

## Method

The study (hypothesis, analyses, exclusion criteria, and sample size justification) was preregistered at https://aspredicted.org/yr8h-nxj4.pdf. The raw data and analysis script are available via the Open Science Framework (OSF) at: https://osf.io/yvj6a/overview?view_only=9b3776c6a93445f693af558e0b693372.

### Participants

Forty-five native speakers of Serbian (mean age: 30 years, range: 23–35; 20 female), residing in Serbia, participated in the experiment. This sample size yielded 1,800 observations per condition, which exceeds the 1,600 observations recommended by Brysbaert and Stevens (2018) to capture small effects in masked priming experiments. Participants were recruited through Ipsos Strategic Marketing Belgrade and were paid for their participation in accordance with company standards. Given that Serbian readers commonly use both Latin and Cyrillic, participants were eligible only if they resided in Serbia and reported daily Cyrillic use. Participants who did not meet the criteria regarding their place of residence and use of the Cyrillic alphabet were not allowed to continue participating. All participants reported normal (or corrected-to-normal) vision and no dyslexia or language-related disorders. They signed an informed consent form before the start of the experiment. The experiment was approved by the Ethics Committee of the University of Valencia in accordance with the guidelines of the Declaration of Helsinki.

### Materials

We selected 240 words from the Serbian Wordlex corpus (Gimenes & New, 2016). Of these, 60 contained each of the letters Л, H, Љ, and Њ. We categorized Л and H as simple letter forms, and Љ and Њ as complex letter forms, yielding 120 words in each set. Words ranged from five to nine letters in length (*M_simple* = 7.22 [*SD* = 1.36], *M_complex* = 6.97 [*SD* = 1.29], *p* = 0.14), had a mean Zipf frequency of 4.67 (*M_simple* = 4.70 [*SD* = 0.52]; *M_complex* = 4.64 [*SD* = 0.44], *p* = 0.33), and had a mean OLD20 of 1.62 (*M_simple* = 1.65 [*SD* = 0.47]; *M_complex* = 1.60 [*SD* = 0.38], *p* = 0.33), computed from *srLex* (Ljubešić et al., 2016). Thus, the simple- and complex-letter target sets were closely matched on the main lexical variables relevant to lexical decision. The critical letters never appeared at the beginning or at the end of the word, and no word contained both simple and complex letter types.

We used a Latin-square design to distribute the three prime conditions (always lowercase prime–UPPERCASE target) across three lists: (1) identity (e.g., кoнaц–КOHAЦ; кoњицa–КOЊИЦA); (2) visually similar, in which the critical letter was replaced by its opposite-form counterpart, yielding a pseudoword prime (e.g., кoњaц–КOHAЦ; кoницa–КOЊИЦA); and (3) visually dissimilar, in which the critical letter was replaced by a visually unrelated letter (e.g., кoдaц–КOHAЦ; кoдицa–КOЊИЦA). For the purposes of lexical decision, we also generated 240 pseudowords using the UniPseudo generator (New et al., 2024). The pseudowords were derived from the word targets, contained the critical letters, and were subjected to the same prime-target manipulation as the word stimuli.

### Procedure

The experiment was programmed in JavaScript using the *jsPsych* package (De Leeuw et al., 2023; Version 7) and hosted on the Cognition.run platform (https://www.cognition.run/) (for validation of online masked priming, see Angele et al., 2022). The entire experiment, including instructions and stimuli, was presented in Serbian Cyrillic. Additionally, to encourage task engagement, participants were informed that their performance would be tracked, and that accuracy would be periodically checked. If accuracy fell below 90%, they were warned that the experiment would be aborted.

Each trial in the lexical decision task began with a mask of hashtags (e.g., #######) presented for 500 ms, followed by the prime (lowercase, 50 ms), and then by the target (uppercase, presented for up to 2,000 ms or until response). The mask, prime, and target were presented in the same font size in a monospaced typeface (Courier New), so that each character occupied the same character cell on the screen. Responses exceeding the timeout were coded as errors. Participants were instructed to press the key “K” if they thought that the target was a word, or “D” if it was not. The instructions emphasized both speed and accuracy, while the prime was not mentioned. The experiment started with 24 practice trials in which participants received feedback (“Correct!”; “Incorrect!”; “Too slow!”). This was followed by 480 experimental trials, including eight possible breaks where an accuracy message was displayed. Participants were randomly assigned to one of the three counterbalancing lists. The presentation of the stimuli was randomized for each participant. The average time to complete the experiment was approximately 20 min.

### Data analysis

As is standard in lexical decision experiments, the analyses were conducted only on word targets. Incorrect responses, responses faster than 250 ms, and responses exceeding the 2,000-ms response deadline were excluded from the response time (RT) analysis; responses exceeding the deadline were coded as errors. This resulted in an overall removal of 2.78% of word trials. No additional upper RT cutoff was applied.

We used the brms package (Bürkner, 2017) in R (R Core Team, 2025) to fit Bayesian generalized linear mixed-effects models to the RT and accuracy data. In both models, the fixed effects were priming condition (identity vs. similar vs. dissimilar) and letter form (simple [Л/H] vs. complex [Љ/Њ]; coded as −0.5 vs. 0.5). The baseline levels were “similar” for priming condition and “simple” for letter form. We used the default priors in brms. For the RT data, we used the ex-Gaussian distribution, whereas for the accuracy data, we used the Bernoulli family (correct responses = 1, incorrect = 0). We specified the maximal random-effects structure justified by the design, with by-subject random intercepts and slopes for priming condition, letter form, and their interaction, and by-item random intercepts and slopes for priming condition. Each model was run for 5,000 iterations across four chains, with 1,000 warm-up iterations. Bayesian (generalized) linear mixed-effects models provide posterior estimates and 95% credible intervals (CrIs). We report posterior means and 95% CrIs and base our interpretation on the direction and magnitude of the effects.

## Results

**Fig. 1:**
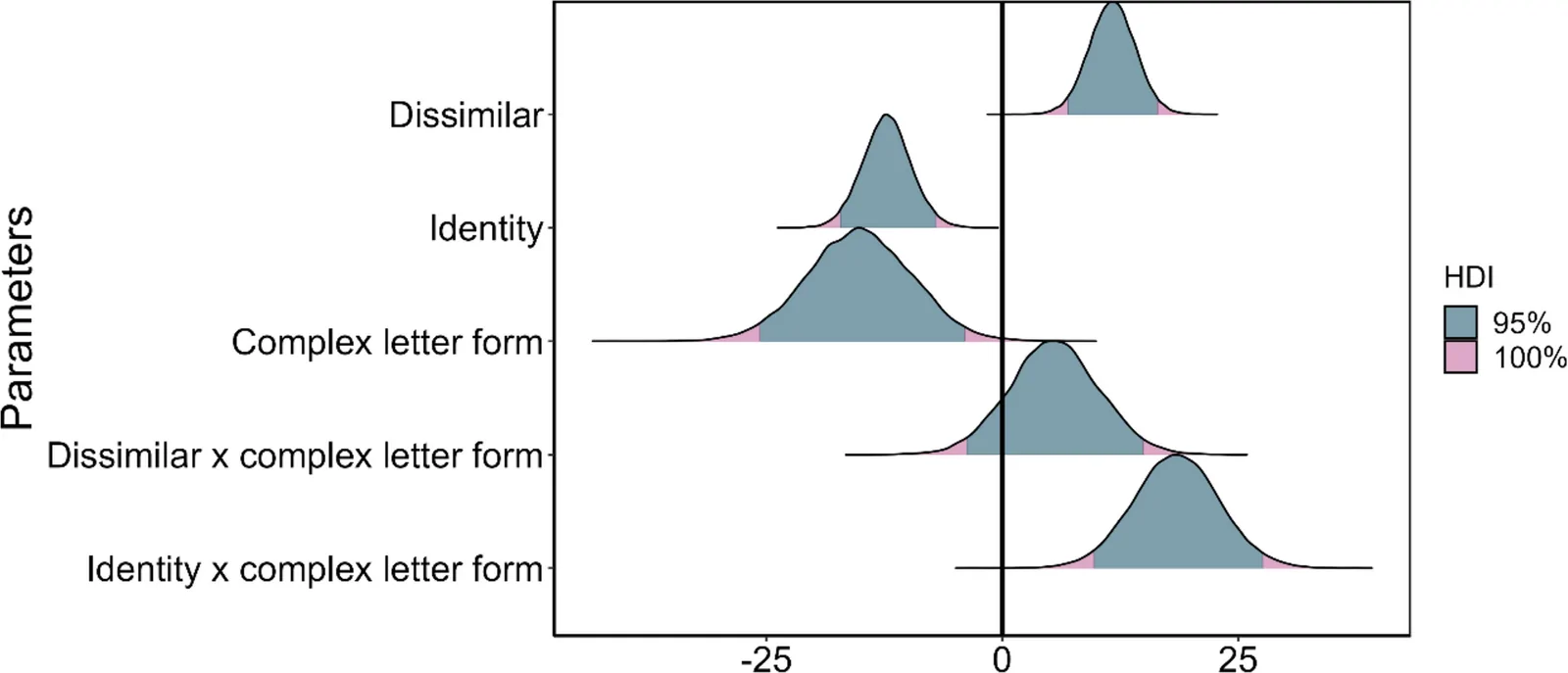
Posterior distributions of the response time model. Highest Density Interval (HDI) legend shows the correspondence of the colored areas to the credible interval percentage. The reference condition for prime-target relationships was the Similar condition

Table 1 shows the mean RTs and error rates in each condition. The posterior RT distributions from the model fit are shown in Fig. 1.

**Table 1:** Mean correct response times (ms) and percent error rates for words. Standard error in brackets

	Identity		Similar		Dissimilar	
	RT	Error rate %	RT	Error rate %	RT	Error rate %
Л/H	681.76 (4.35)	1.78 (0.31)	706.62 (4.63)	2.89 (0.39)	723.38 (4.63)	2.5 (0.37)
Љ/Њ	695.15 (4.77)	2.72 (0.38)	693.77 (4.55)	2.33 (0.36)	714.38 (4.73)	4.39 (0.48)

### Similar versus identity

Responses were faster when preceded by an identity prime than when preceded by a visually similar prime (*b* = −12.25, *SE* = 2.58, 95% CrI [−17.29, −7.12]). Critically, this identity priming effect interacted with letter form (*b* = 18.45, *SE* = 4.55, 95% CrI [9.53, 27.41]): for simple targets (Л/H; i.e., in the feature-addition condition, where the visually similar prime contained the extra feature), the advantage of the identity condition over the similar condition was approximately 25 ms, whereas for complex targets (Љ/Њ; i.e., in the feature-omission condition, where the visually similar prime lacked a feature), the advantage of the identity condition was negligible (approximately 1 ms).

The accuracy model showed no main effects, but it did reveal an interaction between the two factors (*b* = −0.83, *SE* = 0.37, 95% CrI [−1.58, −0.12]), indicating a larger advantage of the identity condition for the simple words than for complex target words.

### Similar versus dissimilar

We also found evidence for an advantage of the similar relative to the dissimilar condition (*b* = 11.64, *SE* = 2.43, 95% CrI [6.87, 16.40]). There was no clear evidence for the interaction with letter form (*b* = 5.47, *SE* = 4.76, 95% CrI [−3.87, 14.84]), indicating that the visual-similarity effect was of comparable magnitude for simple and complex targets (16 and 20 ms, respectively). The accuracy data showed some evidence for an interaction, but given the ceiling effect in accuracy and the small size of the effect, we do not interpret it further.

Thus, identity priming was modulated by letter form. For simple targets (e.g., КOHAЦ), adding a feature in the prime (кoњaц) incurred a reliable processing cost relative to the identity condition. In contrast, for complex targets (e.g., КOЊИЦA), omitting a feature in the prime (кoницa) produced latencies that were indistinguishable from the identity condition. Thus, the present findings indicate that the asymmetry in visual-similarity priming effects is not confined to detached diacritical marks but also arises with letters that differ by an integrated visual feature.

## Discussion

The present experiment examined whether the addition–omission asymmetry previously reported with diacritical letters reflects something specific to diacritics, or a more general property of early orthographic coding. Serbian Cyrillic provides a particularly informative scenario because the critical contrast is carried by an integrated stroke within a single letter (e.g., the consonants Л/Љ and H/Њ), rather than by a detached diacritical mark. The results were clear. When the prime omitted a feature present in the target (кoницa → КOЊИЦA), priming was indistinguishable from identity priming. In contrast, when the prime added a feature absent from the target (кoњaц → КOHAЦ), priming was reliably reduced. Thus, the same asymmetry previously reported with diacritics (e.g., Benyhe et al., 2023; Marcet et al., 2020; Perea et al., 2020) emerges with integral letter features.

One possible concern is that primes containing the more complex letters Љ/Њ may be harder to encode under brief presentation than primes containing Л/H. However, this interpretation is not supported by the data. Visually similar primes produced an advantage over dissimilar primes for both simple and complex targets, and the size of this advantage was comparable across the two letter types (16 and 20 ms). Thus, primes containing Љ/Њ were sufficiently processed to generate partial orthographic overlap, suggesting that what matters is not prime complexity per se, but whether the prime provides compatible or conflicting feature-level evidence. A related concern is that the simple letters Л/H are slightly more frequent in Serbian than their complex counterparts Љ/Њ. A similar issue arises in experiments with diacritics, where letters with diacritics tend to be less frequent than their non-diacritic counterparts (e.g., see Perea et al., 2020). However, a simple letter-frequency account does not predict the direction of the critical interaction. If anything, the greater frequency of Л/H should lead to generally stronger evidence for the simple-letter forms. Thus, it does not explain why Л/H primes were as effective as identity primes for Љ/Њ targets, whereas Љ/Њ primes were less effective for Л/H targets. Moreover, visually similar primes facilitated responses relative to visually dissimilar primes for both target types, and the magnitude of this effect was comparable for simple and complex targets.

A phonological account is equally unlikely. In the present materials, the visually similar substitution necessarily changed the identity of the critical phoneme in both directions (e.g., н ↔ њ; л ↔ љ; see also Marcet et al., 2020, for a similar asymmetry with n ↔ ñ; see also Bae et al., 2025, for evidence in Korean Hangul). If phonological mismatch were driving the effect, both substitution types should have incurred a comparable cost. However, they did not, suggesting that the asymmetry lies in the visual-orthographic code rather than in phonological overlap. More specifically, a prime missing the distinctive feature of a complex letter (e.g., н for њ) can still function as compatible evidence for that letter, whereas a prime containing the distinctive feature of a complex letter (e.g., њ for н) provides strong counter-evidence for the simple letter. This yields an asymmetric similarity relation at the level of early orthographic coding, regardless of whether the critical feature is realized as a detached diacritic or as an integral part of the letter form.

Thus, the present findings argue against a strictly diacritic-specific account of the addition–omission asymmetry in visual-similarity effects (cf. Perea et al., 2020). Instead, the asymmetry appears to reflect a more general property of feature-based orthographic coding, whereby the presence of a feature may carry stronger bottom-up evidence than its absence. This interpretation is compatible with the noisy-channel model (see Kinoshita et al., 2021), but it should not be taken as unique to this model. It is also consistent with feature-based accounts of letter perception in which omission yields a degraded but still compatible match, whereas addition introduces conflicting evidence and hence stronger competition at the letter level. This latter view fits naturally with the model proposed by Rey et al. (2009), in which feature detectors activate letter units via excitatory mappings and lateral inhibition between letter units serves to resolve ambiguity. According to this account, omission primes preserve compatibility with the target letter, whereas addition primes activate a competing letter representation by virtue of the extra feature. The present data extend this logic beyond diacritics to letter pairs that differ by an integrated letter feature.

More generally, our findings reinforce the need for computational models of visual word recognition to explicitly model the interface between layers of visual features and abstract letter units. Current models of visual word recognition focus on how combinations of letter units drive lexical activation but have been much less explicit about how featural letter evidence is converted into those letter units in the first place (e.g., see Davis, 2010; Snell, 2025). The present findings constrain how this mapping should be implemented, revealing an asymmetry in the weighting of feature presence versus feature absence. An informative extension would be to examine Serbian readers in both scripts. Serbian Latin includes letters with detached above-letter marks (e.g., č, š, and ž) as well as digraphs for some of their Cyrillic counterparts, including the contrasts examined here. A within-language comparison of detached diacritical features and integrated visual features would provide a direct test of how these feature types are weighted during early orthographic processing.

In sum, the present results show that feature-level encoding is a core component of orthographic processing, highlighting the need for models of word recognition to articulate explicitly the mapping from visual features onto abstract letter units. In particular, the asymmetry between feature addition and feature omission appears to be a general asymmetry of early orthographic coding rather than a peculiarity of diacritical marks.

## Data Availability

Raw data, analyses scripts, and stimuli are available via the OSF at: https://osf.io/yvj6a/overview?view_only=9b3776c6a93445f693af558e0b693372; preregistration is available at: https://aspredicted.org/yr8h-nxj4.pdf.

## Code availability

The code of the experiment is available via the OSF at: https://osf.io/yvj6a/overview?view_only=9b3776c6a93445f693af558e0b693372.
